# Supplementary material for: Delineating clinical and developmental outcomes in STXBP1-related disorders
Source: Brain. 2023 Nov 28;146(12):5182–97. doi: 10.1093/brain/awad287 (PMC10689925; doi:10.1093/brain/awad287)
Supplement: awad287_Supplementary_Data [file awad287_supplementary_data.pdf]

Supplementary Material

Supplementary Methods. Validated outcome measures in *STXBP1*

Supplementary Methods. Longitudinal seizure frequency forecasting

Supplementary Methods. Comparative effectiveness analysis and treatment response

Supplementary Methods. Framework for virtual clinical trials

Supplementary Table 1 Demographics of the Children's Hospital of Philadelphia (CHOP) cohort and Ciitizen Natural History Registry

Supplementary Table 2 Correlations between age at epilepsy onset and severity of developmental outcome

Supplementary Table 3 Single decision tree, showing the branching of subgroups based on epilepsy characteristics or predictors for unpredictable seizure trajectories

Supplementary Figure 1 Reconstructed epilepsy histories across the cohort

Supplementary Figure 2 Distribution of *STXBP1* diagnosis by year

Supplementary Figure 3 Stratification of epilepsy trajectories by recurrent variants in *STXBP1*

Supplementary Figure 4 PDMS-2 measure of age equivalent scores and percentiles

Supplementary Figure 5 Milestone acquisition across epilepsy onset subgroups

Supplementary Figure 6 Developmental outcomes stratified by seizure severity

Supplementary Figure 7 ASM response across epilepsy syndromes

Supplementary Methods: Validated outcome measures in *STXBP1*

The need to provide interventions in early childhood to affect motor development is well established in children with CP. During typical childhood development, gross motor skills generally plateau by 4–7 years of age,[1] while GMFCS levels remain stable throughout childhood.[2] However, the early years of life are an exception with less stability in GMFCS classifications before the age of 2 years.[3]

While outcome measures and classification scales developed for children with CP may be used in *STXBP1*-related disorders,[4] future longitudinal research is needed to determine if their trajectory of development is like children with the broader diagnosis of CP, especially if motor outcome is to be used to determine the effectiveness of future clinical trials.

References

1. Beckung E, Carlsson G, Carlsdotter S, Uvebrant P. The natural history of gross motor development in children with cerebral palsy aged 1 to 15 years. Dev Med Child Neurol. 2007;49(10):751–6
2. Palisano RJ, Cameron D, Rosenbaum PL, Walter SD, Russell D. Stability of the gross motor function classification system. Dev Med Child Neurol. 2006;48(6):424–8.
3. Gorter JW, Ketelaar M, Rosenbaum P, Helders PJ, Palisano R. Use of the GMFCS in infants with CP: the need for reclassification at age 2 years or older. Dev Med Child Neurol. 2009;51(1):46–52
4. MacLennan AH, Lewis S, Moreno-De-Luca A, et al. Genetic or Other Causation Should Not Change the Clinical Diagnosis of Cerebral Palsy. *J Child Neurol*. Jul 2019;34(8):472-476. doi:10.1177/0883073819840449

Supplementary Methods: Longitudinal seizure frequency forecasting and quantification of unpredictability

We developed a framework for longitudinal seizure frequency forecasting to characterize epilepsy progression and predictability. Standardized seizure histories were captured using a previously published scale derived from the Pediatric Epilepsy Learning Health System (PELHS)-championed framework for seizure severity, and seizure frequencies were grouped in the following categories: multiple daily seizures (>5 per day, SF score = 5), several daily seizures (2–5 per day, SF score = 4), daily seizures (SF score = 3), weekly seizures (SF score = 2), monthly seizures (SF score = 1), and no seizures (SF score = 0). All individuals with complete seizure histories in the first year of life (n=78 individuals) were included in the seizure frequency forecasting analysis.

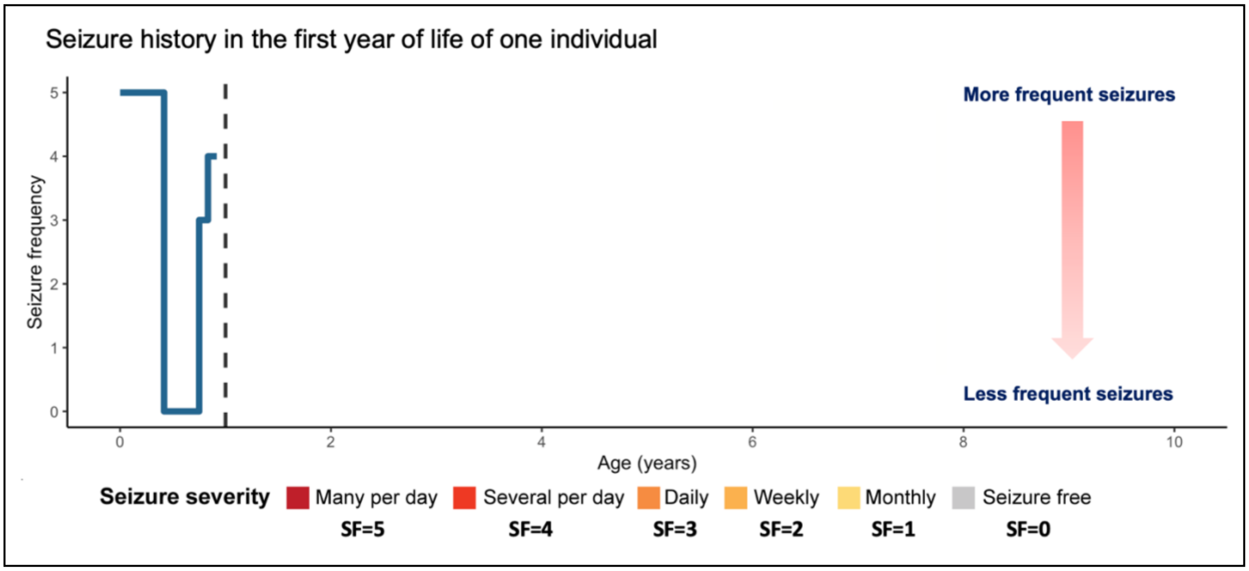

We compared seizure frequencies across monthly time intervals between all combinations of individual patient pairs during the first 12 months of life, when seizures are the most prominent in *STXBP1*. Based on the framework for semantic similarity analysis to measure phenotypic resemblance using clinical features,[1,2] we derived a complementary method to measure phenotypic similarity based on epilepsy histories, using monthly seizure frequency captured in the PELHS framework as comparisons (**see below**).

First, we calculated the information content (IC) of each seizure frequency (SF) category in each month (e.g., SF score = 3 during Month 8 of life). The IC was defined as the -log2 of the frequency of individuals in the SF category during the respective month. For example, if 20% of the cohort had at least daily seizures during the 8th month of life, then the IC for SF score = 3 was defined as -log2(0.20) = 2.32. Accordingly, the higher the frequency of individuals with a certain seizure frequency in a specific month, the less informative that seizure frequency for the respective month. In contrast, if only 5% of individuals had daily seizures at 7 years of life, an SF score = 3 would be more informative, and thus weighed more when deriving phenotypic resemblance.

To quantify phenotypic resemblance between two individuals, we summed the minimum IC overlap of each month across all overlapping months. As in the semantic similarity analysis, the rationale was to derive a higher similarity measure for individuals with more rare and thus more informative, or distinguishing seizure frequencies. For example, if two individuals had a less frequent seizures across many months, the derived similarity measure would be higher than two individuals with the same seizure

frequency across fewer months and higher than two individuals with a seizure frequency that was more common in the overall cohort. To correct for varying observation times between patient pairs, we adjusted the cumulative IC score by dividing by the number of overlapping months of comparison for each respective patient pair.

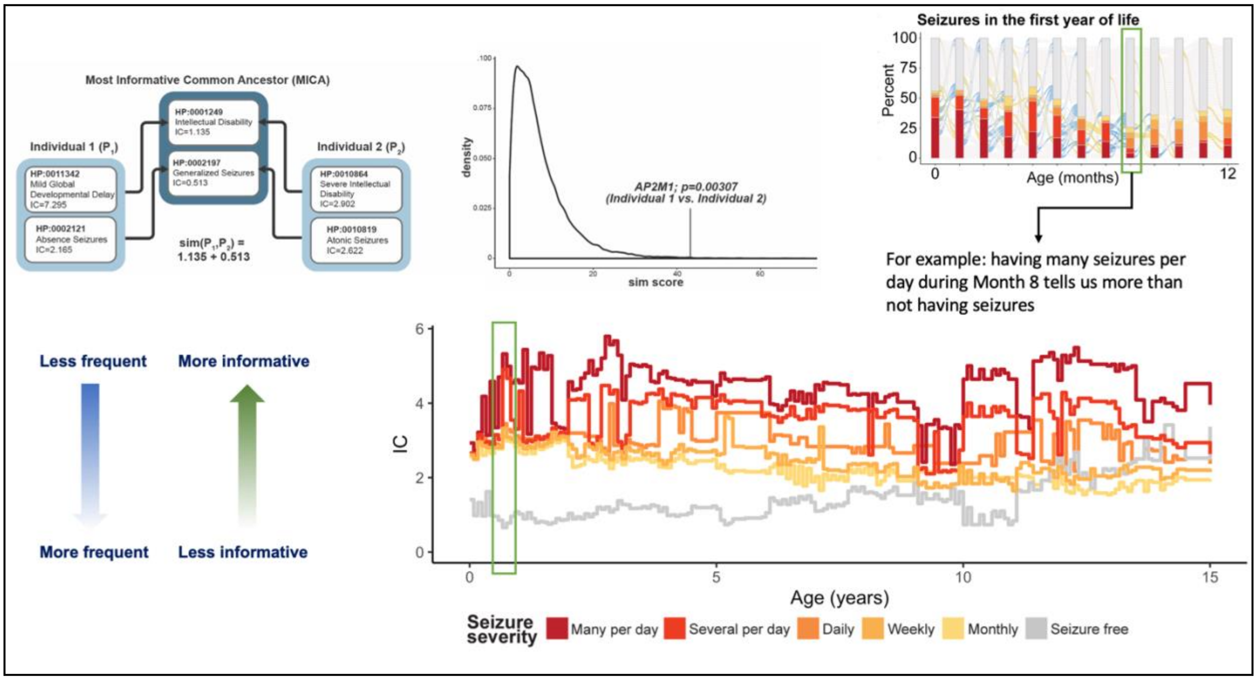

After deriving phenotypic similarity scores for all patient pairs in the first year of life, for each individual, we then identified 10 distinct individuals in the cohort that were the most similar phenotypically based on seizure frequencies across the age span, which was defined as that individual's reference cohort.

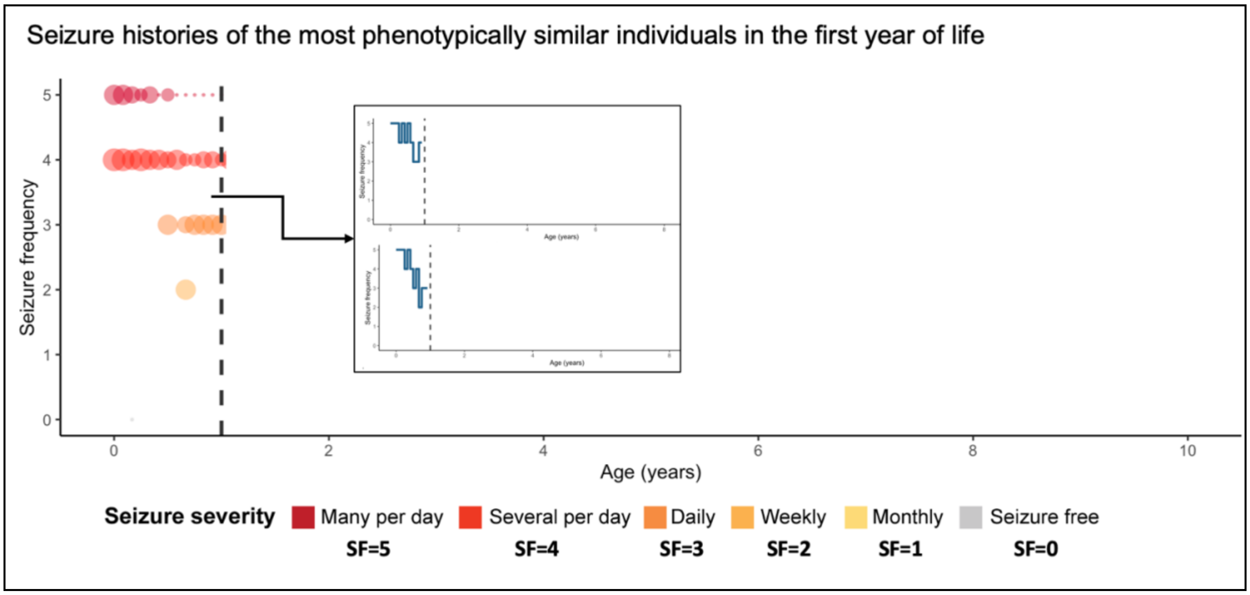

Then, with the known epilepsy histories in the reference cohort, including seizures after 12 months of life, we predicted each individuals' epilepsy trajectory after the first year, taking the median of the distribution of seizure frequencies for each month in the reference cohort, representing a distribution of likelihood of possible seizure frequency outcomes, as the predicted trajectory.

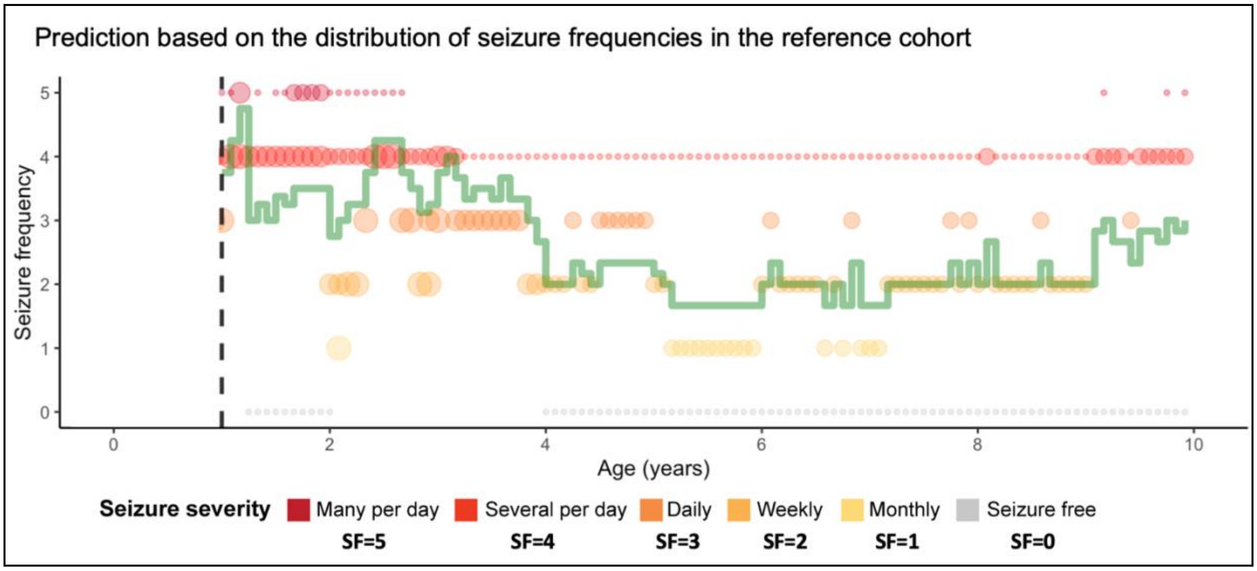

The predicted (example shown below in green) and actual seizure trajectories (example shown below in blue) after the first year of life was compared and assessing the cumulative difference enabled us to characterize subgroups defined by a high or a low difference between the forecasted and actual seizure frequencies, which we defined as a measure of epilepsy unpredictability. The grouping was performed using the k-means algorithm. For each individual, forecasted seizure frequencies were compared to a distribution of randomly generated seizure frequencies and permutation testing of 100,000 for each individual estimation allowed us to evaluate whether the predicted trajectory was better than chance.

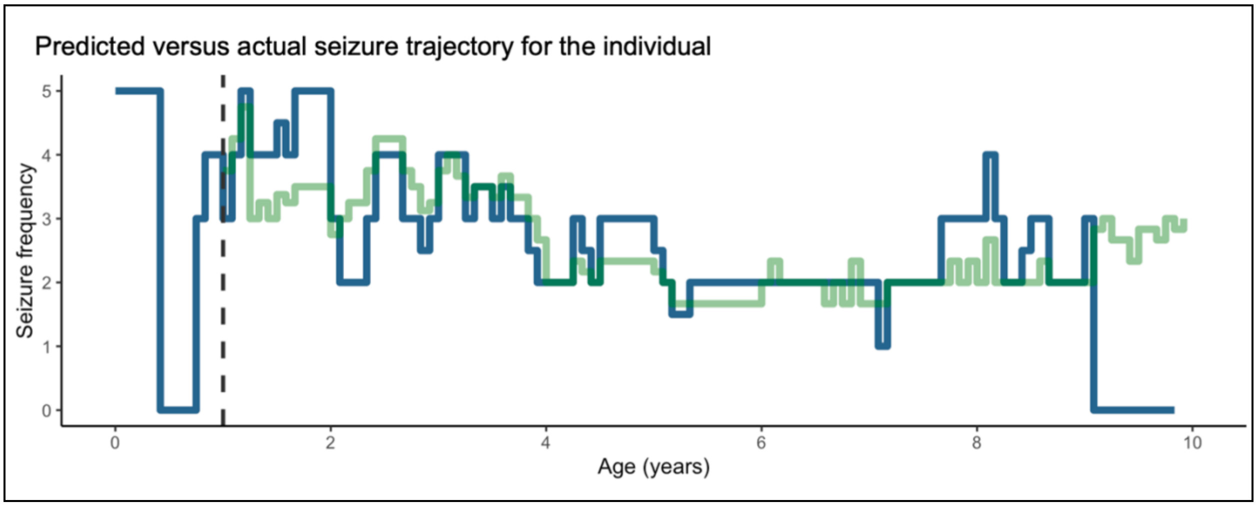

References

1. Galer PD, Ganesan S, Lewis-Smith D, et al. Semantic similarity analysis reveals robust gene–disease relationships in develop- mental and epileptic encephalopathies. Am J Hum Genet. 2020; 107(4):683–697.
2. Helbig I, Lopez-Hernandez T, Shor O, et al.; GRIN Consortium. A recurrent missense variant in AP2M1 impairs clathrin-mediated endocytosis and causes developmental and epileptic encephalopathy. Am J Hum Genet. 2019;104(6):1060–1072.

Supplementary Methods: Comparative effectiveness analysis and treatment response

We measured medication efficacy through short-term treatment response in addition to long-term treatment response in three analyses:

1. For the first analysis, we analyzed the relative effectiveness of treatment strategies in seizure reduction, indicated by a decrease in seizure frequency by any degree. We compared months in which periods of seizure reduction coincided with certain ASMs compared to months of no response, which was defined as seizures worsening indicated by an increase in seizure frequency by any degree, or continuous active seizures with the same frequency. We did not include periods of seizure freedom in the category of no response to treatment (i.e., the seizure frequency score had to be above 0).

2. For the second analysis, we analyzed the relative effectiveness of treatment strategies in either seizure reduction or maintaining seizure freedom. We compared seizure reduction or having consecutive months of being seizure-free versus worsening of seizures, indicated by an increase in seizure frequency.

3. For the third analysis, we analyzed the relative effectiveness of treatment strategies in maintaining seizure freedom only, comparing periods of seizure freedom indicated by consecutive months of no seizures versus worsening of seizures as no response to treatment. We did not include periods of seizure reduction in this analysis.

Associations are presented as odds ratios with 95% confidence intervals, correcting for multiple comparisons using a False Discovery Rate (FDR) of 5%.

Supplementary Methods: Framework for virtual clinical trials

Given the heterogeneity of seizures in *STXBP1*-related disorders, we aimed to identify time windows during which a treatment effect would have the highest probability of being detected in a clinical trial. We derived a framework for virtual clinical trials, randomly sampling 20 individuals with ongoing seizures and simulated a 6-month and 12-month period of 10%, 15%, and 20% seizure reduction.

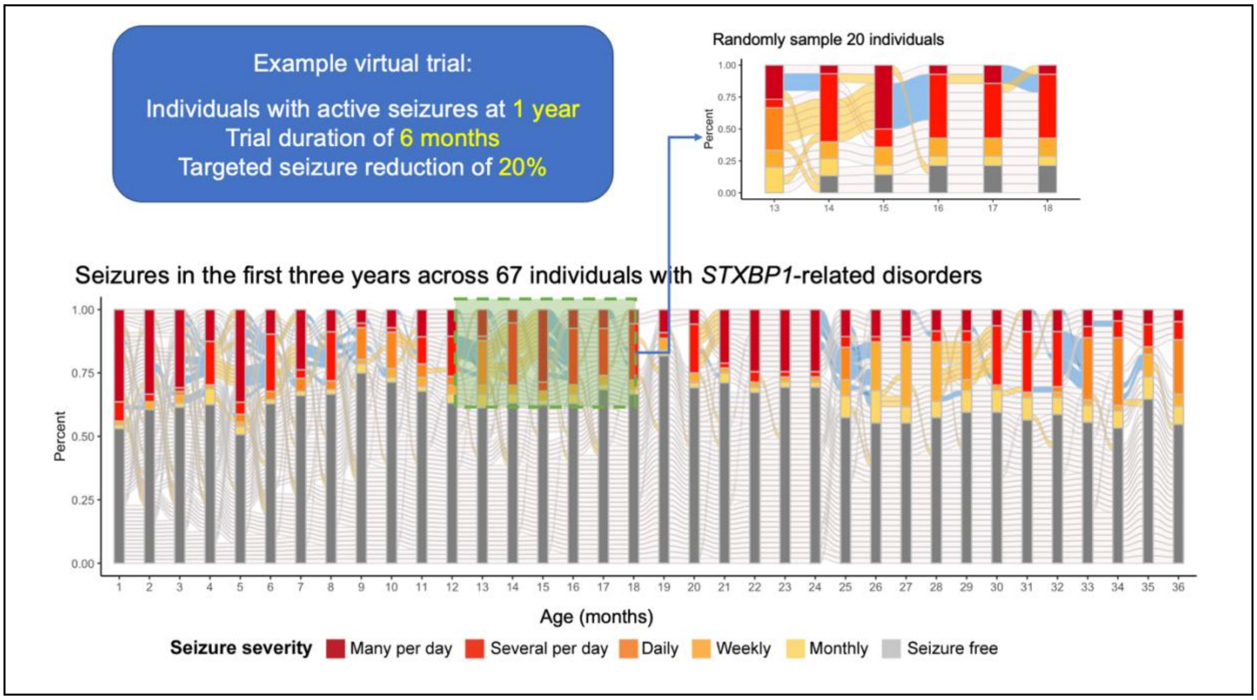

The percent reduction in seizures was calculated based on the cumulative sum of seizure frequencies at the start of the window for the sampled cohort, and the simulated treatment effect across each trial window was performed by decreasing seizure frequencies across the trial period for this cohort to create a synthetic treatment cohort.

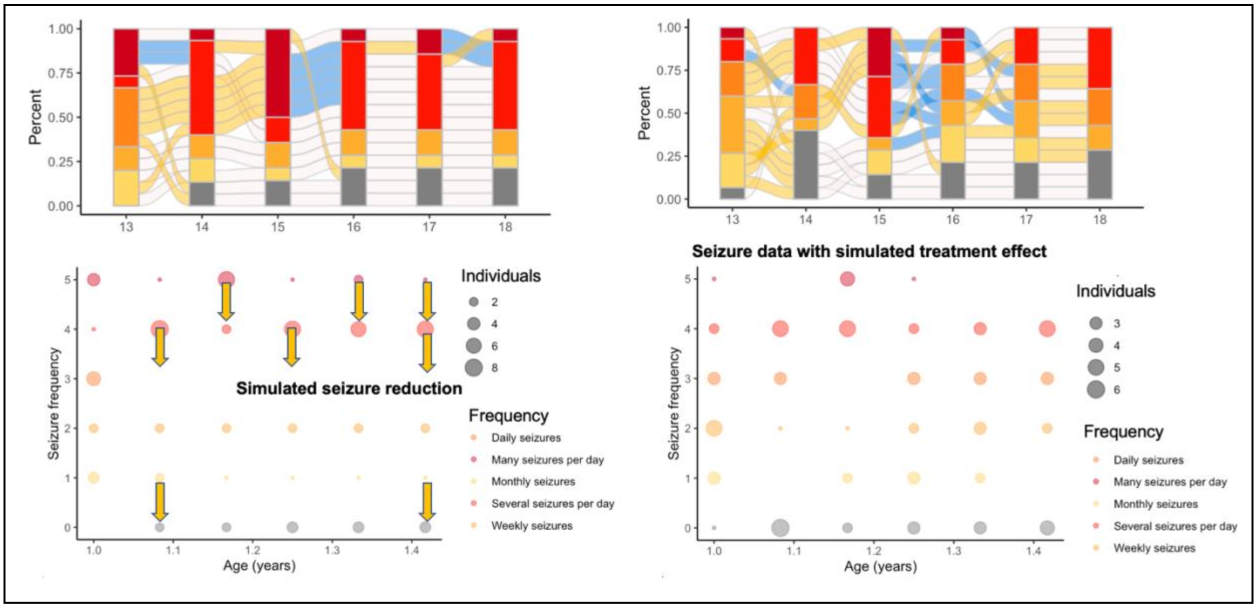

We used the synthetic control method to evaluate the significance of the simulated effect, comparing the distribution of seizure frequencies following simulated reduction of seizures against the observed, natural history distribution of frequencies in the sampled individuals using the Wilcoxon rank sum test.

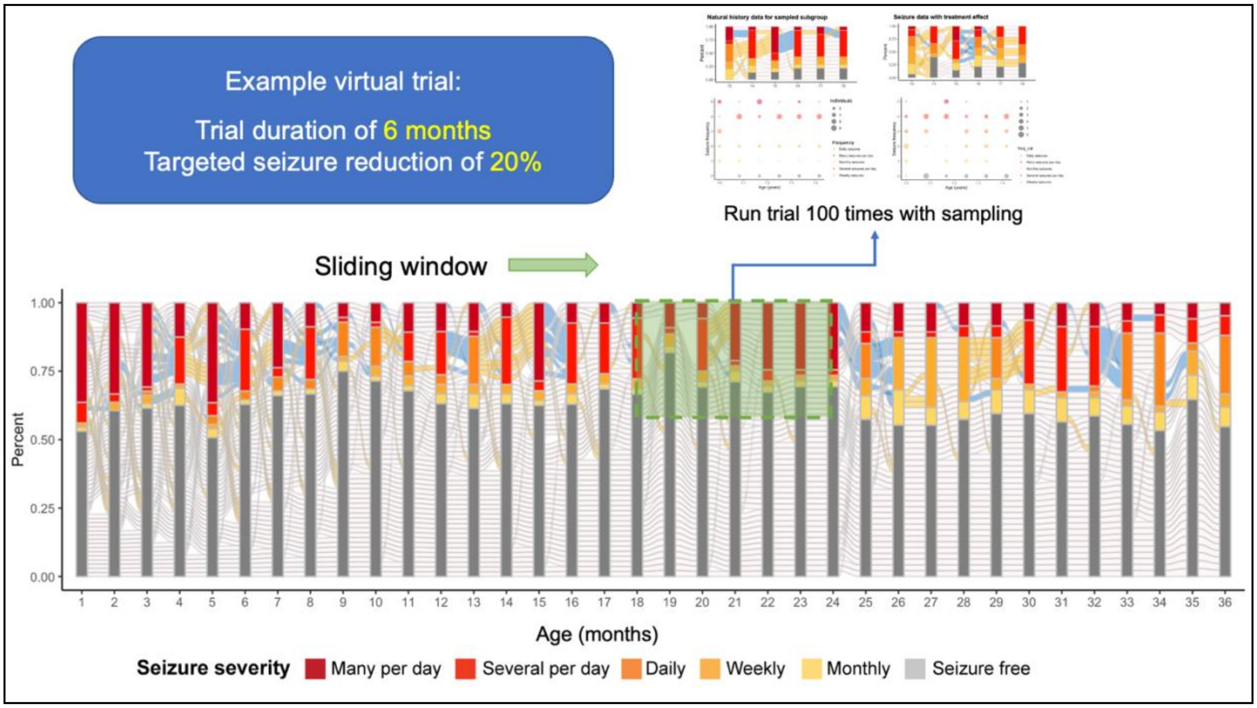

We ran 1,000 simulated trials for each month across the age span, shown above an example 6-month trial starting at 18 months of life. For each trial window, the Observed Frequency of Trial Success (OFTS) was defined as the proportion of trials out of 1,000 in which a significant effect could be detected. For analyses across seizure types, we chose to include the trial duration (6-month versus 12-month period) and targeted seizure reduction (10%, 15%, 20%, or 50%) based on the distribution of OFTS that resulted in the widest range, spanning from poor probability of trial success to high probability across the age span. This approach enabled us to identify optimal windows during which a treatment response would most likely be observed in a real-world trial when using seizure frequency as the primary outcome measure.

Supplementary Table 1 Demographics of the Children's Hospital of Philadelphia (CHOP) cohort and Ciitizen Natural History Registry

	Children's Hospital of Philadelphia (n=104)	Ciitizen Natural History Registry (n=58) [a]
**Demographics**		
Male	57 (54.8%)	24 (41.4%)
Female	47 (45.2%)	34 (58.6%)
**Age distribution**		
Age at assessment	Median 4.9 years<br>IQR 2.6 – 12.8 years<br>Range 3 months – 44.2 years	Median 5.5 years<br>IQR 3.1 – 101 years<br>Range 5 months – 34.7 years
**Genetic spectrum**		
Missense variants	46 (44.2%)	34 (58.6%)
PTV/del [b]	55 (52.9%)	23 (39.7%)
In-frame indels	3 (0.03%)	1 (0.02%)

[a] In our study, clinical documentation analyzed from the Ciitizen Natural History Registry only included milestone acquisition data. The Ciitizen database includes curated clinical documentation from medical records across multiple hospitals and providers where a patient had been seen and received clinical care. In brief, this data was reviewed and verified by trained medical curators, mapped to standardized clinical terminology, and de-identified curated data was then made available to academic researchers.
[b] Protein-truncating variants (PTV)/dels included splice sites, frameshifts, and whole and partial gene deletions.

Supplementary Table 2 Correlations between age at epilepsy onset and severity of developmental outcome

Developmental measure [a]	Individuals assessed/available data	Association/correlation [b]
**Milestone acquisition**		
Ability to control head posture	63 individuals	P = 0.7
Ability to roll over	69 individuals	P = 0.02 [c]
Ability to grasp	66 individuals	P = 0.1
Ability to sit unsupported	69 individuals	P = 0.1
Ability to initiate words	71 individuals	P = 0.007
Ability to walk	65 individuals	P = 0.002
**Age of milestone achievement**		
Ability to control head posture	5 individuals	-- [d]
Ability to roll over	50 individuals	R = -0.1, P = 0.6
Ability to grasp	28 individuals	R = -0.04, P = 0.9
Ability to sit unsupported	36 individuals	R = -0.1, P = 0.5
Ability to initiate words	12 individuals	R = -0.3, P = 0.4
Ability to walk [e]	30 individuals	R = -0.2, P = 0.2
**Standardized assessments**		
GMFM-66-IS	46 exams	R = 0.2, P = 0.2
PDMS-2	26 exams	R = 0.2, P = 0.4
**Classification scales**		
GMFCS-ER	36 assessments	R = -0.4, P = 0.02
MACS	6 assessments	R = -0.5, P = 0.4
CFCS	16 assessments	R = -0.2, P = 0.5

[a] Abbreviations: Gross Motor Function Measure (GMFM-66-IS); Gross Motor Function Classification System Extended & Revised (GMFCS-ER); Peabody Developmental Motor Scales (PDMS-2); Manual Ability Classification System; (MACS), Communication Function Classification System (CFCS).

[b] Distribution of epilepsy onset in individuals stratified by milestone acquisition (achieved versus not achieved) was performed using Wilcoxon Rank Sum test. Age distribution at which milestones were achieved was assessed using Pearson Correlation Coefficient. Correlation between epilepsy onset and developmental outcomes as measured via validated scales was performed using the Spearman Correlation Coefficient.

[c] Only 8 of 69 individuals did not achieve the milestone of rolling over, limiting interpretation of correlation.

[d] We were unable to assess correlation due to the limited sample size, with all individuals assessed for the specific milestone having neonatal seizure onset.

[e] Only individuals of at least one year of age was assessed.

Supplementary Table 3 Single decision tree, showing the branching of subgroups based on epilepsy characteristics or predictors for unpredictable seizure trajectories.

**Clinical characteristics in the first year of life**	**1**	**2**	**3**	**4**	**5**	**6**	**7**
Ongoing seizures between 7-12 months	No	No	Yes	Yes	Yes	Yes	Yes
Ongoing seizures at 12 months			No	Yes	Yes	Yes	Yes
More than 5 cumulative months with seizures	No	Yes					
Seizures in the first 2 months of life				No	Yes		
Ongoing focal seizures after 4 months of life					No	Yes	Yes
Ongoing infantile spasms after 6 months of life						No	Yes
Probability of unpredictable seizures later in life	0.12	1.00	1.00	0.86	1.00	0.75	0.00
Percent of all individuals	55%	2%	7%	16%	9%	9%	2%

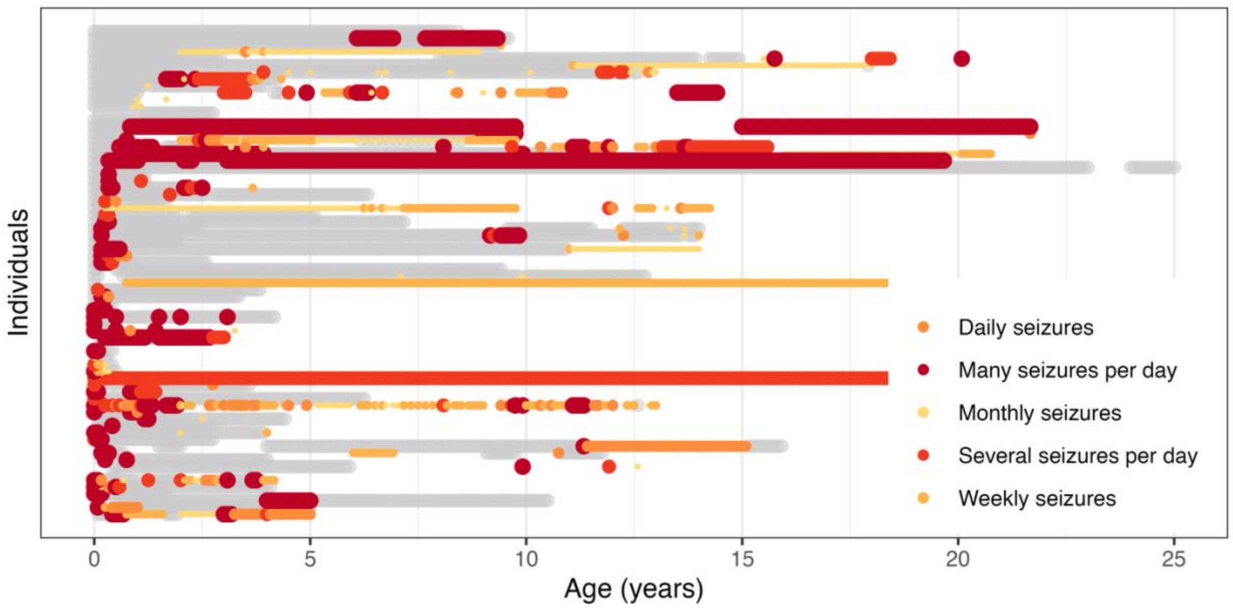

Supplementary Figure 1 Reconstructed epilepsy histories of 72 individuals with seizures. Seizure severity is indicated by ordinal seizure frequency (SF) scores: multiple daily seizures (SF = 5), several daily seizures (SF = 4), daily seizures (SF = 3), weekly seizures (SF = 2), monthly seizures (SF = 1), no seizures (SF = 0). Shown is the pattern of seizure frequency in individuals with epilepsy, regardless of type; individuals are tracked across time, with each row along the y-axis representing a unique individual and the colour representing seizure severity. Grey indicates observation time and periods in which the individual was seizure free.

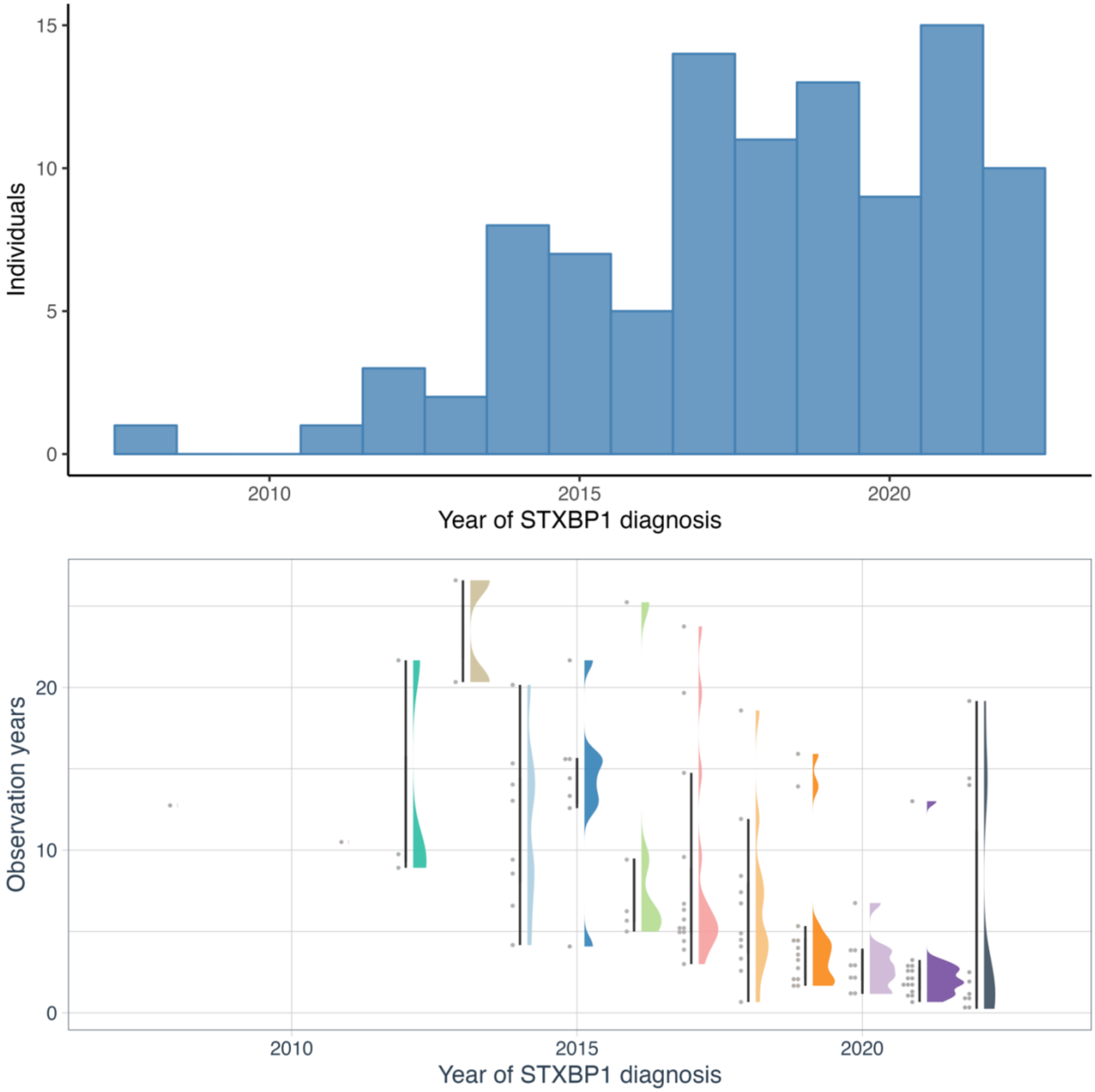

Supplementary Figure 2 Distribution of *STXBP1* diagnoses by year. We mapped the distribution of diagnoses in our cohort by year, showing the first genetic diagnosis in 2008 and most recent in 2022 in our cohort (above), and the range of observation years per individual stratified by year of *STXBP1* diagnosis (below). Data were reconstructed based on clinical histories documented in clinical visits or from available outside medical records, with specificity as reported by the family or clinician.

Supplementary Figure 3 Stratification of epilepsy trajectories by recurrent variants in *STXBP1*

We compared epilepsy severity across the age span in individuals with recurrent missense variants with suspected dominant effects, including p.Arg406Cys/His/Ser (*n*=9), p.Arg292Cys/His/Pro (*n*=8), and p.Arg551Cys/His (*n*=3) versus the remainder of the cohort. Individuals with p.Arg292Cys/His/Pro had slightly higher risk for neonatal seizures (*n*=4, p=0.04) and focal-onset seizures throughout the first two years of life. We also found a significant difference with frequent seizures between 12 and 18 months of life in two individuals with p.Arg551Cys/His, overlapping the period during which seizure remission was observed in >50% of the remainder of the cohort. When grouping the three recurrent variant hotspots (p.Arg406Cys/His/Ser, p.Arg292Cys/His/Pro, and p.Arg551Cys/His, *n*=20), the association was less robust with only an increase in tonic seizures between 7 and 8 months, which was primarily driven by two individuals with p.Arg292Cys/His/Pro variants. This may suggest that unique epilepsy signatures are associated with specific recurrent variants rather than with the broader variant group with suspected dominant-negative effects.

Recurrent variants: p.Arg406Cys/His/Ser, p.Arg292Cys/His/Pro, p.Arg551Cys/His

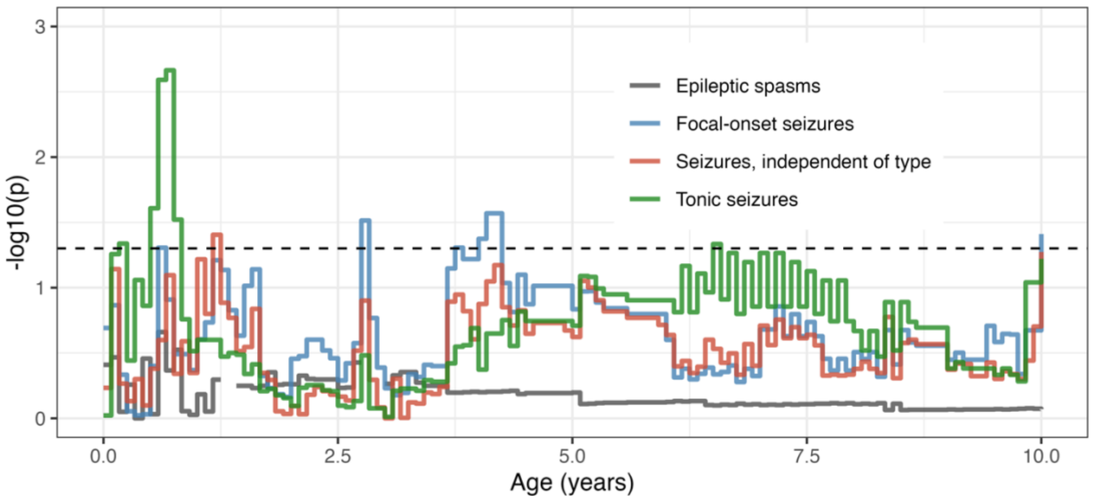

Recurrent variant: p.Arg406Cys/His/Ser

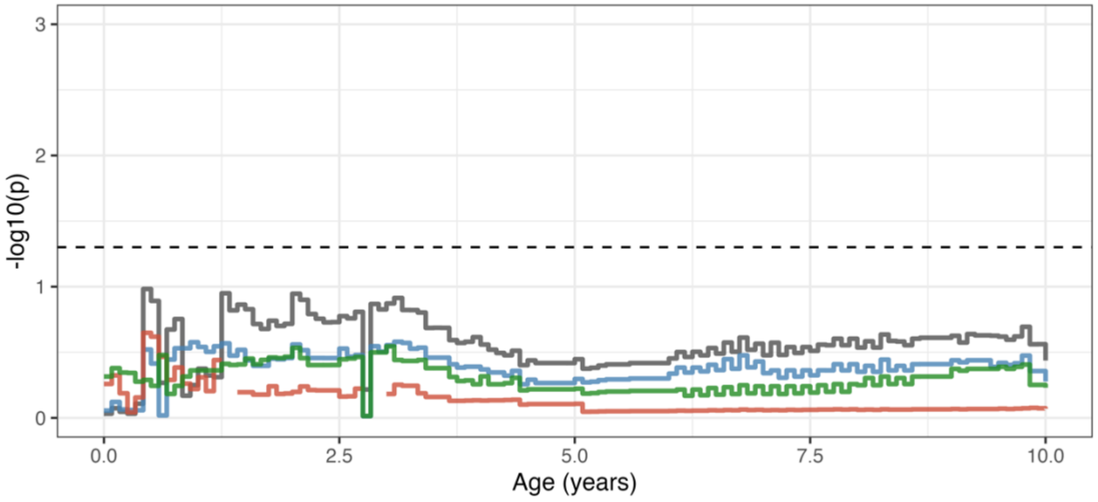

Recurrent variant: p.Arg292Cys/His/Pro

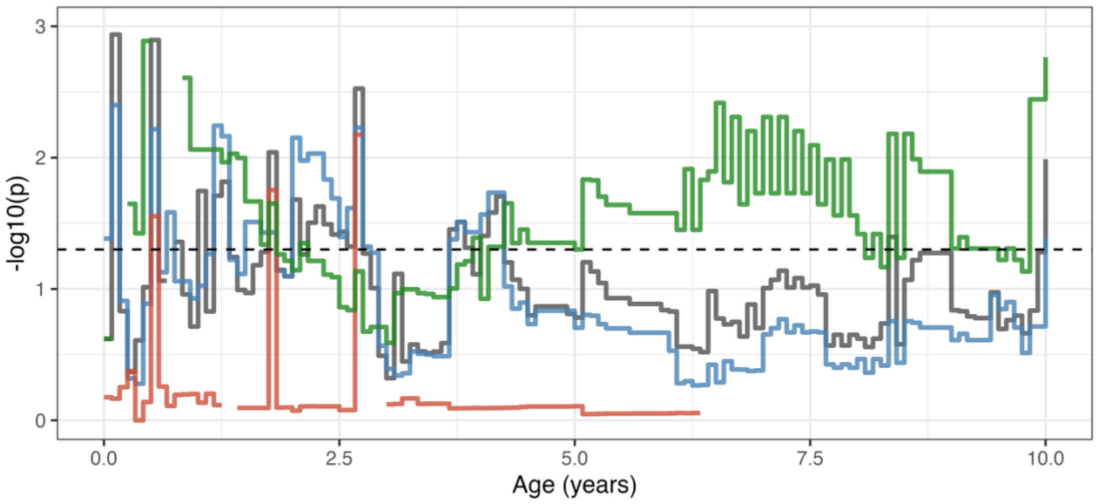

Recurrent variant: p.Arg551Cys/His

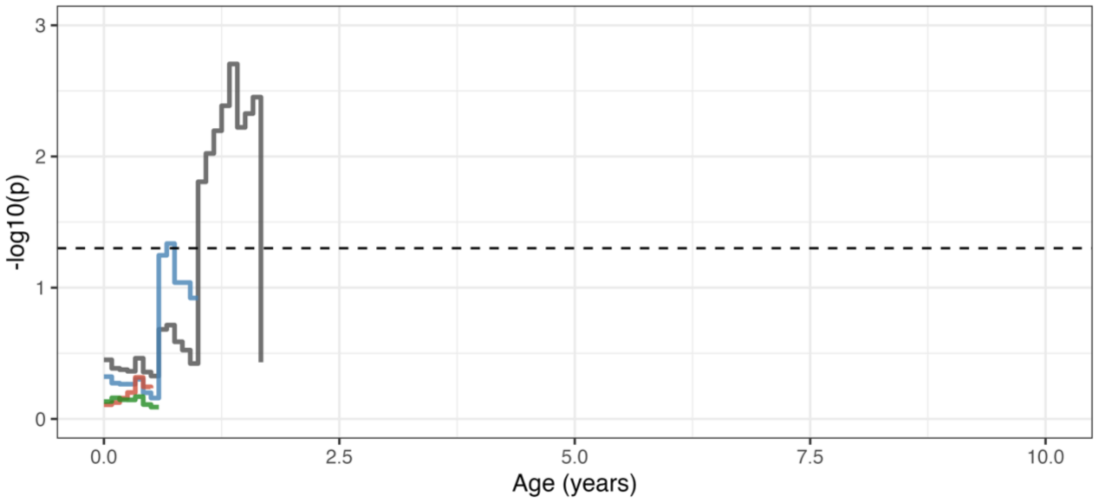

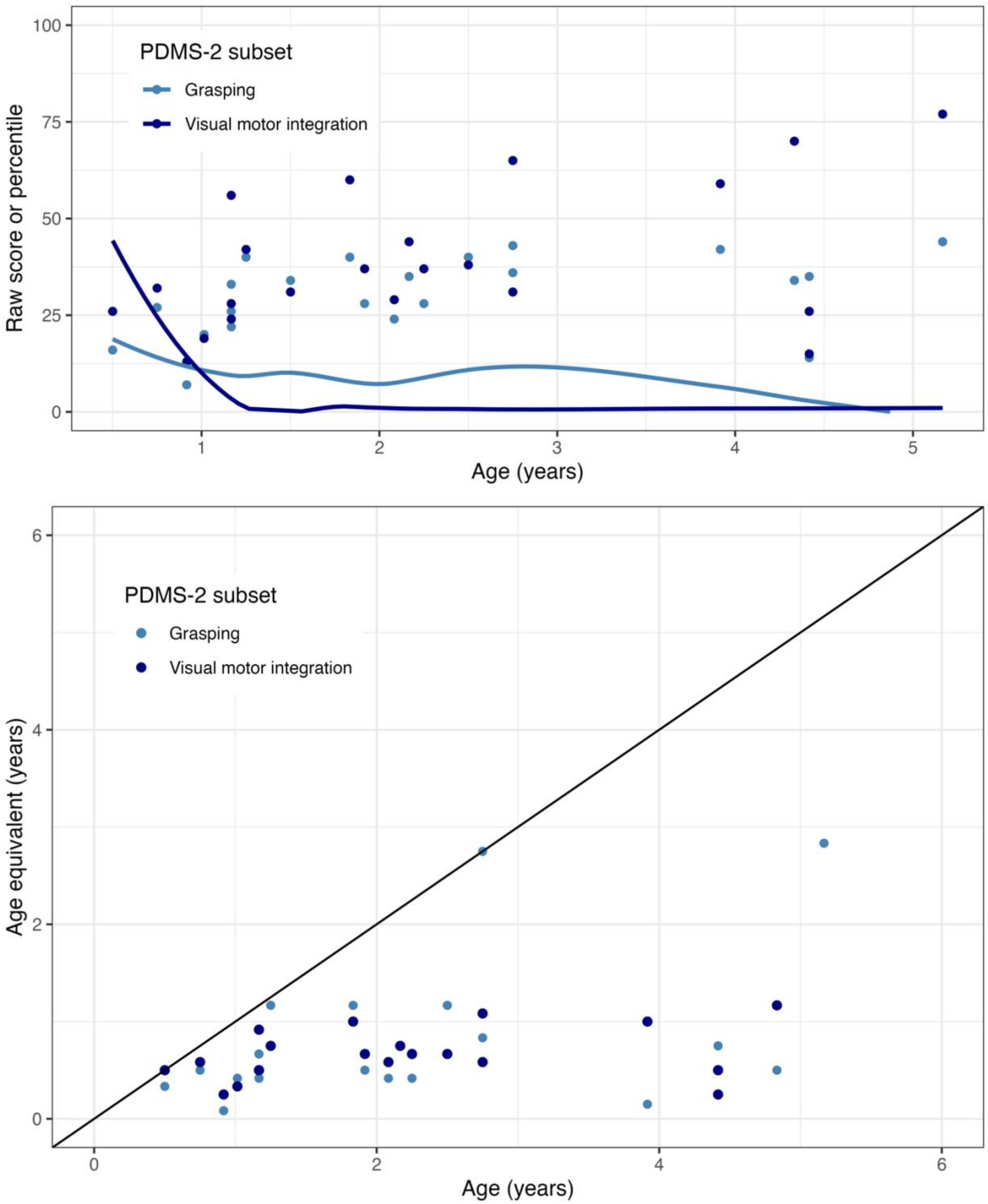

Supplementary Figure 4 PDMS-2 measure of age equivalent scores and percentiles. We assessed the range of PDMS-2 raw scores for grasping and visual motor integration, showing the corresponding percentiles across the age span in reference to typically developing children (above) and age equivalent versus age at assessment (below). Raw scores for the PDMS-2 were used as the outcome metric as the floor effect is observed following the first year of life.

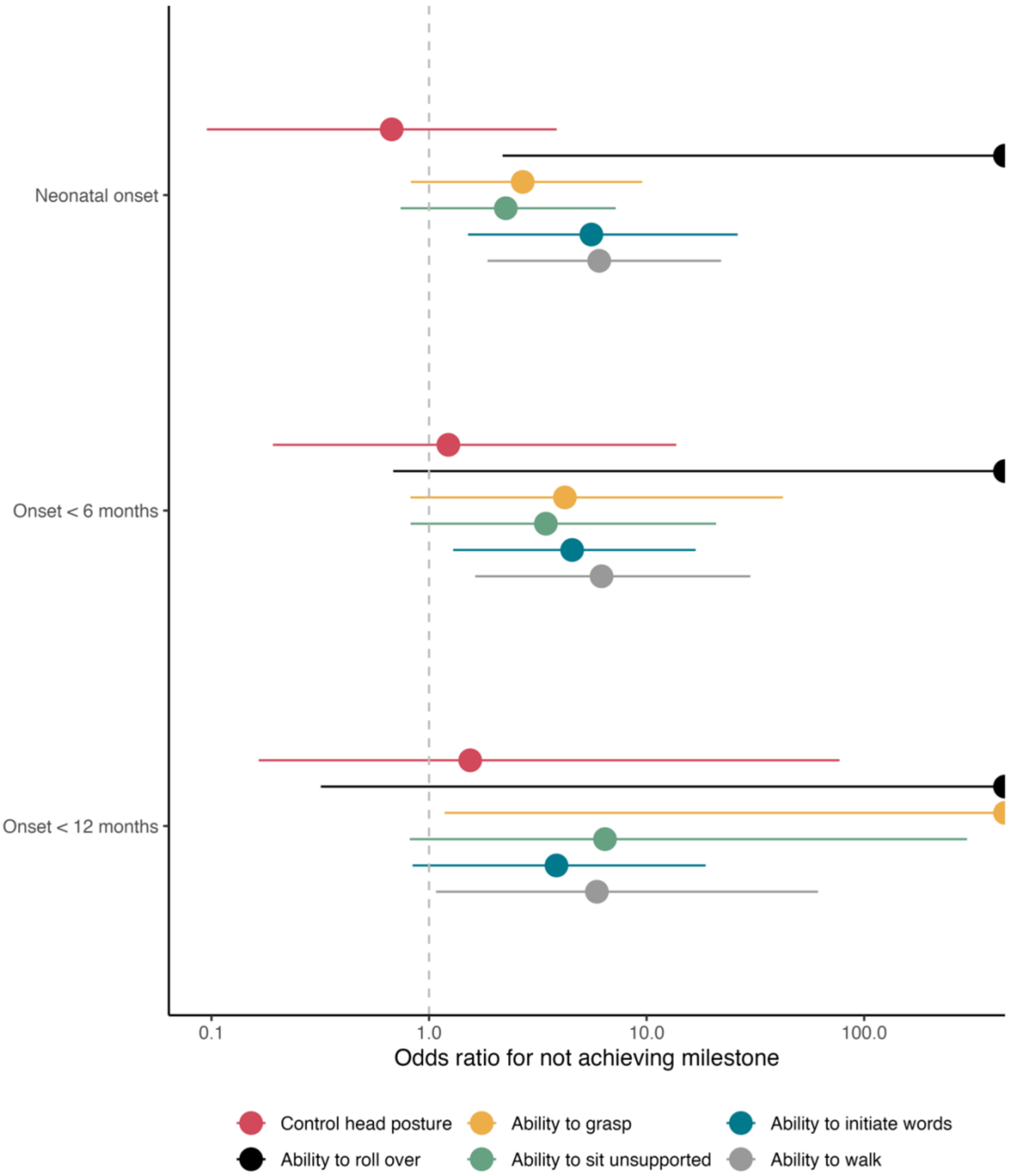

Supplementary Figure 5 Milestone acquisition across epilepsy onset subgroups. We assessed the correlation between developmental milestone acquisition and epilepsy onset, stratified by neonatal seizure onset, seizure onset within the first 6 months of life, and seizure onset in the first year of life. Findings show odds ratios that the respective milestone was not achieved in the epilepsy onset group compared to the remainder of individuals with a later seizure onset. To control for subgroup differences, we found no correlation between age of onset and age at last assessment in the subset of individuals with epilepsy (Pearson correlation coefficient, n = 72 individuals, R = 0.19, p = 0.1); thus, we would not expect to see significant differences in developmental abilities when stratified by age of assessment.

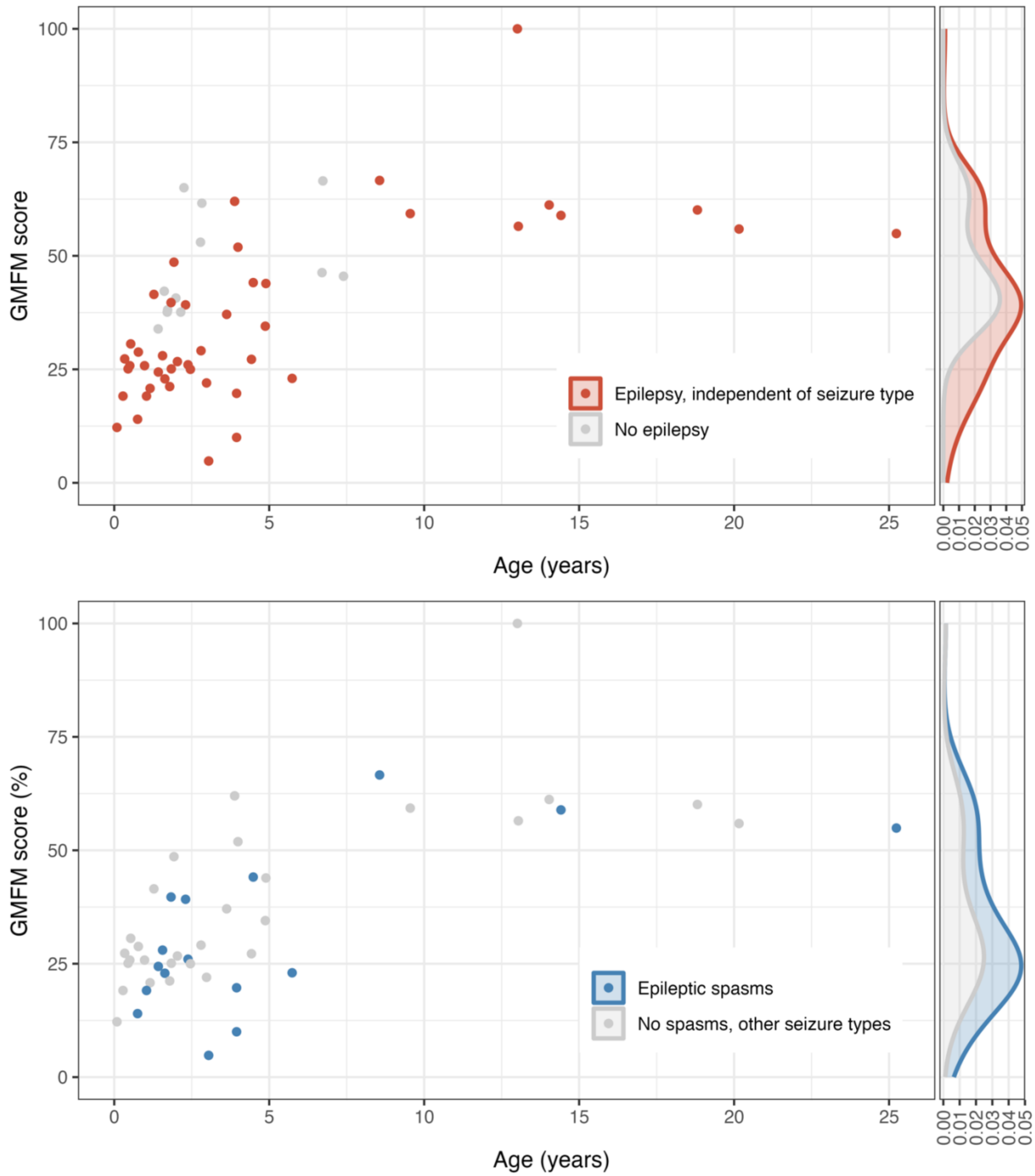

Supplementary Figure 6 Gross motor developmental outcomes across epilepsy subgroups. We assessed the range of GMFM-66-IS raw scores, stratified by individuals with seizures versus without seizures (above) and individuals with spasms versus individuals with other seizure types (below). The Wilcoxon rank sum test was used to determine statistical significance.

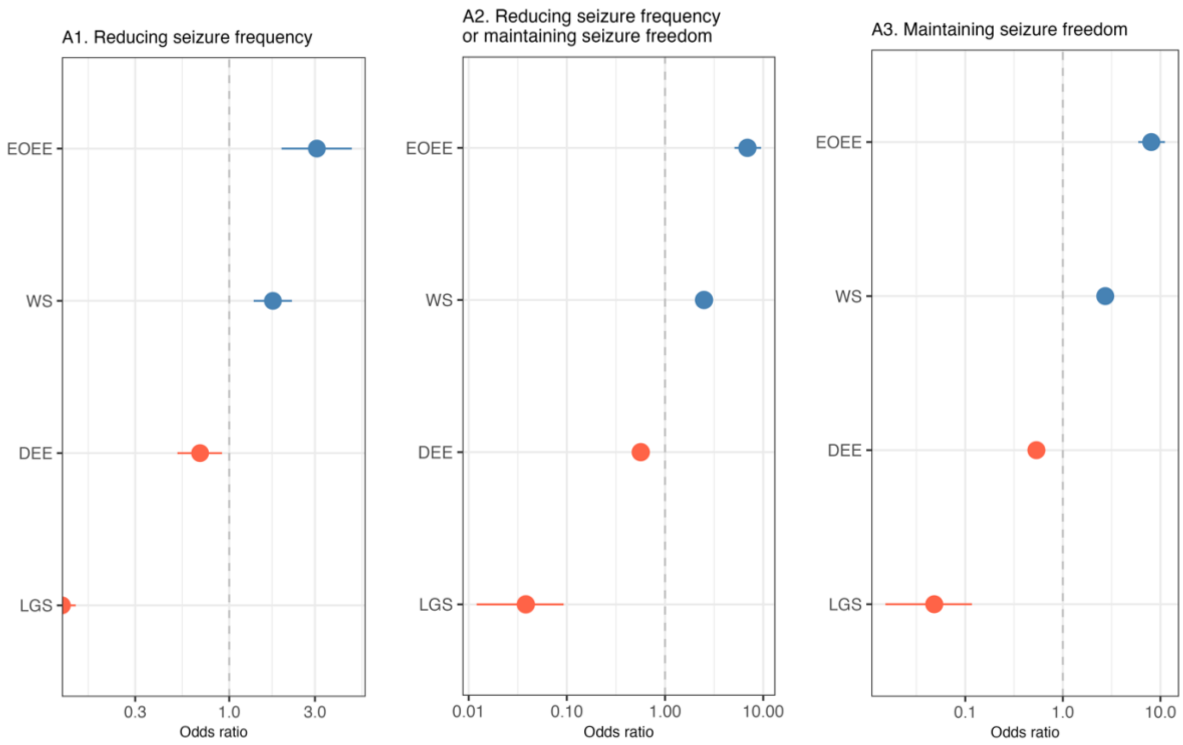

Supplementary Figure 7 ASM response across epilepsy syndromes. Medication response across epilepsy syndromes: West Syndrome (WS), Developmental and Epileptic Encephalopathies (DEE), Lennox-Gastaut Syndrome (LGS), and Early Onset Epileptic Encephalopathies (EOEE). Odds ratios indicate the relative effectiveness of any anti-seizure medications (ASM) or treatment strategy in reducing seizure frequency and/or maintaining seizure freedom.
